# Mesoscopic Structure Conditions the Emergence of Cooperation on Social Networks

**DOI:** 10.1371/journal.pone.0001892

**Published:** 2008-04-02

**Authors:** Sergi Lozano, Alex Arenas, Angel Sánchez

**Affiliations:** 1 ETH Zurich, Swiss Federal Institute of Technology, Zurich, Switzerland; 2 Universitat Rovira i Virgili, Tarragona, Spain; 3 Instituto de Biocomputación y Física de Sistemas Complejos (BIFI), Universidad de Zaragoza, Zaragoza, Spain; 4 Grupo Interdisciplinar de Sistemas Complejos (GISC), Departamento de Matemáticas, Universidad Carlos III de Madrid, Leganés, Spain; 5 IMDEA Matemáticas, Campus de la Universidad Autónoma de Madrid, Cantoblanco, Madrid, Spain; Yale University, United States of America

## Abstract

**Background:**

We study the evolutionary Prisoner's Dilemma on two social networks substrates obtained from actual relational data.

**Methodology/Principal Findings:**

We find very different cooperation levels on each of them that cannot be easily understood in terms of global statistical properties of both networks. We claim that the result can be understood at the mesoscopic scale, by studying the community structure of the networks. We explain the dependence of the cooperation level on the temptation parameter in terms of the internal structure of the communities and their interconnections. We then test our results on community-structured, specifically designed artificial networks, finding a good agreement with the observations in both real substrates.

**Conclusion:**

Our results support the conclusion that studies of evolutionary games on model networks and their interpretation in terms of global properties may not be sufficient to study specific, real social systems. Further, the study allows us to define new quantitative parameters that summarize the mesoscopic structure of any network. In addition, the community perspective may be helpful to interpret the origin and behavior of existing networks as well as to design structures that show resilient cooperative behavior.

## Introduction

The emergence and survival of cooperation in adverse environments has been, for a long time, a challenging problem for scholars in disciplines as diverse as biology, sociology or economics [1]–[3]. While some partial answers have been advanced in the last forty years [4], cooperation among unrelated individuals is far from understood. Social dilemmas, situations in which individual rationality leads to situations in which everyone is worse off, are a prominent example of this conundrum [5]. Within the general framework of evolutionary game theory, which is particularly well suited to study this problem, the Prisoner's Dilemma (PD) is a paradigmatic setting to capture the paradox of altruism persistence against short-term benefits of egoism. In this game two players choose between cooperation (C) or defection (D), the payoffs for the two actions being as shown in the following (Table 1).

**Table 1:** Payoff matrix of the Prisoner's Dilemma.

	C	D
**C**	1	0
**D**	*b*	ε

Payoffs received by the row player when plays against the strategy in the column. The relation among the payoffs is: *b*>1>*ε*>0.

Relations between different possible payoffs follow the rule *b*>1>*ε*>0, that immediately poses the dilemma: While the rational choice is to defect, it leads to a highly inefficient outcome as compared to that obtained by two cooperators. In other words, a decision that should be good for the individual leads to a poor result from the global (group, social) viewpoint. This is the most stringent social dilemma in so far as to defect is a dominant strategy: “Softer” dilemmas (stag-hunt, snowdrift [5]) require to coordinate or anti-coordinate with the other player's choice, but there is not a dominant option. We focus here on the PD because it represents the situation in which cooperation is more difficult and, therefore, its origin and stability is more problematic.

Among the plethora of studies devoted to this issue, a particularly important and fruitful one is the modeling of the population as a set of non-rational, learning agents that interact locally [6]–[11] (see [12] for a very recent review). Locality is introduced in the model through a network on which agents are placed. These agents then play the game only with their neighbors (in neighborhoods that can be defined in different ways) instead of interacting with all other agents. Learning is introduced through imitation: after a round of games has been carried through the whole lattice, agents look at their neighbors and choose the strategy that has led to the highest payoff before proceeding to the next round of games. With these two ingredients, namely locality and imitation, it is generally observed [6], [11], [13] that states in which a sizeable part of the population cooperates emerge (at least for values of *b* not too close to 2), the mechanism for this emergence being the formation of clusters of cooperators that can successfully outcompete defectors.

Naturally, the question arises as to whether this mechanism for the emergence of cooperation appears also in real social networks [14]. As a first step to answer this question, some authors have focused their interest on the influence of certain macroscopic structural features that have been observed in real networks on the evolution of cooperation, such as the small-world phenomenon [15] or the scale-free character of the degree distribution [16]. A general conclusion of this research is that the inhomogeneity of the degree distribution plays a central role on this issue, and that it may favor the emergence of cooperation. However, none of these studies deals either with true social network substrates or with more specific mesoscopic structures, in particular, the community structure present in many real social networks. The motivation on the use of real social substrates relies on the fact that these networks present structural characteristics that are often not reproduced by general network models. Although it is true that real social network structures must be a particular instance of all possible synthetic cases, instead of exploring the myriad of possibilities within the model space, it seems more convenient to use real social networks to get insight as to which structural features one should look at for advancing in the understanding of the evolution of cooperation.

To our knowledge, there is only one paper about the PD on real social networks [17], but its point of view is dynamical and unrelated to the present one. Therefore, our research is a first attempt to understand the relevance of considering empirical social networks as a topological support for the local interactions in the framework of imitation models. The analysis of the results allows us to claim that it is mandatory to consider structural features of networks at the mesoscale (basically its community structure) to understand the arising of cooperation. We also propose a model network construction algorithm that synthetizes different mesoscopic structures into networks, and that allows crosschecking this claim on *in silico* substrates. Furthermore, the model can be used to build networks with specific cooperative properties, which can later be employed in the design of organizations. We have thus completed a research cycle going from the observation of the behavior of cooperation on real networks to the modelling and application of our conclusions through the identification of the most relevant features of the problem.

## Materials and Methods

### Datasets

For our research we have used two social substrates obtained by sampling real relational data. We have chosen these substrates instead of other social network data available, such as the IMDB network for actor collaboration in movies or the scientific collaborating networks, because their links are defined through true personal exchanges. In contrast, these other public data are bipartite networks, where links are defined by joining the collaboration framework (movies, research projects, articles, etc.) which does not necessarily imply mutual interactions. Our first substrate is a social network obtained from the email traffic between members of University Rovira i Virgili (in Tarragona, Spain; email network from now on), where nodes represent individual email addresses and undirected links between two nodes indicate bidirectional communication (at least one email in each direction) [18]. Our second real social substrate consists of nodes representing users of the “Pretty-Good-Privacy” encryption algorithm (PGP network, from now on), while links trace trust relationships between those persons who sign each other's public keys [19]. For a comparison of some of their statistical properties see Table 2.

**Table 2:** Statistical properties of e-mail and PGP networks.

Network	Ref.	N	P(k)	〈C〉	r
email	[18]	1133	∼exp (-*k*/9.2)	0.25	0.078
PGP	[19]	10680	∼*k* ^∧^(−2.63) if *k*<40	0.26	0.238
			∼*k* ^∧^(−4.0) if *k*>40		

N is the number of nodes of the giant component of the network considering only those links that are bidirectional (indicating mutual acquaintance between nodes). P(k) is the degree distribution, i.e., the histogram of the number of nodes with a give degree k (analytical expressions are best fits to the data using least squares method). 〈C〉 is the average clustering coefficient and indicates the fraction of existing triangles between each node and its neighbours in the network, and r stands for the assortativity coefficient, that measures the tendency of nodes of high degree to link between them, r>0, or with nodes of lower degree, r<0 [43].

### Dynamics

Our simulations of the PD over all the networks (both email and PGP, as well as on the models to be introduced below) follow strictly the rules in [6], [8], namely:

- Initial strategies of agents are assigned randomly with the same probability to be C or D (we have checked that other choices for the initial fraction of C or D lead to similar results, see Figure S1).
- The game is played between each pair of neighbors, and payoffs are accrued according to b>1, and *ε* = 0 although we checked that its value (being small, e.g. 0.01) does not affect the results, see Figure S2.
- Accumulated payoffs of all agents are computed by adding up the results of the games with their neighbors in the present turn.
- In the next round, every agent imitates the strategy of the most successful agent in her neighborhood (randomly selected if there are two or more agents with the same payoff), after which payoffs are reset to zero.

While the networks we use are obtained from experimental measurements and, as such, are given, there are different options for the learning rule of the agents we place on the network. We chose to stick to the (unconditional) imitation rule described above for a a number of reasons. From the methodological viewpoint, imitation allows a direct comparison to other studies, such as [6], [8] while, on the other hand, its deterministic character makes its numerical study much more amenable. Importantly, for global interactions learning by imitation ends up in global defection, and hence cooperation in a local model can not be due solely to this learning rule. From the theoretical viewpoint, it is clear that other rules, such as best-reply, will lead straightforwardly to a fully defecting population even with local interactions. It can be argued that imitation is too simple a rule but, as discussed in [9], there are several reasons why agents may fail to recognize they are in a dilemma situation, which would lead them to defection; another reason for the use of imitation is as a mode of economizing behavior [20]. From the experimental viewpoint, there are several reports that indicate that imitation is commonly used by humans [21]–[23]. Finally, imitation can be justified in psychological terms by looking at how confirmation and disconfirmation of beliefs are carried out [24] and has been also proposed as a relevant force to drive the evolution towards economic equilibrium [25]. Specific aspects where the use of other learning mechanisms can change our results will be discussed below (see Conclusions).

Finally, we note that the update rule for strategies is synchronous, i.e., all agents update their strategy at the same time, proceeding to a new round of the game subsequently. Changing to a non-synchronous update is known to have non-trivial consequences [7], [26]. However, non-synchronicity is difficult to deal with in general, as the particular way to introduce it comes dictated by the application of interest and different procedures lead to different results; that is why it has been considered only rarely in the framework of evolutionary game theory, and only in very simple and arguably arbitrary ways [7], [8]. Note also that the rule is based on the total payoff accumulated by every player, which obviously makes hubs more influential than nodes with very few links. One could think of using the average payoff (i.e., the payoff divided by the degree). While this choice would make nodes more equivalent, it requires the players to be aware of very much information about their neighbors, and this information is more difficult to obtain than the absolute payoff (or some estimate of it).

## Results

**Figure 1:**
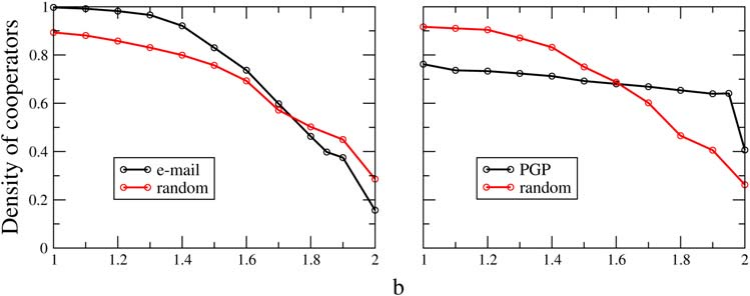
Evolution of cooperation in real social networks. Black lines: Density of cooperators as a function of b, obtained by numerical simulations on the email (left) and PGP (right) networks. Red lines: Density of cooperators on random networks generated from the original ones by a rewiring procedure that preserves the degree distribution(see text). The equilibrium densities of cooperators have been obtained by averaging 500 generations, after a transient time of 750 generation steps. Each point corresponds to an average over 1000 independent simulations with 50% cooperators and defectors as the initial condition.

Let us begin by examining the results of simulations of the PD on real social networks as a function of the temptation parameter *b*. In Fig. 1 we plot the final density of cooperators on the two cases addressed here, the email network and the PGP network. The first remarkable feature of these plots is the high level of cooperation attained even for large values of *b* on both networks, as compared to the results on regular lattices [6], [8], [11] with the same imitation dynamics. The cooperation levels are not as high as those reported by Santos *et al.* [16], [27], [28] on scale free networks, although in their simulations the dynamics is stochastic, and therefore a direct comparison can not be made. In this regard we also want to stress that the two networks we are analyzing can not be considered scale-free: The email network has a clear exponential distribution of degrees, and the PGP network presents two regions with a clear crossover from a power law behavior with exponents −2.63 (for degree k<40) and −4 (for degree k>40) strongly indicating a bounded degree distribution.

Nevertheless, the crucial result arising from Fig. 1 is that the dependence of the level of cooperation on the temptation parameter *b* is very different for both networks. As we may see from the plots, the cooperation level on the email network is a decreasing function of *b*, going from values very close to unanymous cooperation for b≈1, to about a 15% for *b* close to 2. On the contrary, the PGP network presents an almost constant cooperation level, with a variation of a 10% at most in all the range of *b* values, except for *b* = 2. These results inmediately lead to the conclusion that there is no typical behavior of the cooperation level on true social networks, at least in the framework of the PD with imitation dynamics or learning.

The above conclusion is further reinforced by noting that the cooperation level in each network changes in a very different manner when their original structure is distorted. To this end, we have compared the results on the two networks with their randomized version preserving the degree of each node, carried out through a rewiring process [29]. The process, that consists of repeatedly choosing at random two nodes and exchanging one neighbor of each node (also selected randomly), destroys correlations between nodes (and in particular the community structure we will discuss below). Figure 1 shows clearly that playing the game on the real networks and on their randomized versions gives rise to opposite behaviors: On the email network cooperation reaches extremal values, higher than the random case when *b* is close to 1, and lower when *b* is close to its maximum limit of 2. On the contrary, on the PGP network cooperation is higher on the random version for low values of the temptation *b*, and worse for higher values. Remarkably, the cooperation level in the random versions of the two networks is very similar, and close to those reported in [27] for the configuration (random) model, although it must be kept in mind that the dynamics is different in the latter case; interestingly, this does not seem to induce large differences in behavior in this respect.

Our two examples, email and PGP, do not seem to fit in any of the categories previously reported in the literature for the behavior of the PD, which implies that the macroscopic (global, statistical) similarities between both topologies (see Table 2) are not determinant for the opposite behaviors observed. Furthermore, the fact that randomization, while preserving the degree distribution, drives the behavior of the two networks to the same general pattern, indicates that neither the whole network nor individual agents provide the clue to understanding our observations. Therefore, in order to gain insight on this problem, we must consider an intermediate, mesoscopic organizational level as the possible source of the explanation for the dramatic differences observed in the original systems. This in turn requires a deeper analysis of the structure of both networks, which is what we subsequently do.

### Communities

As a first attempt to understand networks at a mesoscopic level, we propose to focus on their community structure. Community structure is a common feature of many networks: Communities can be qualitatively defined as subgraphs with dense connections within themselves and sparser ones between them. A quantitative definition of communities is introduced as the partition of a network that optimizes the quality function known as modularity: 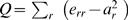 where *e_rr_* are the fraction of links that connect two nodes inside the community *r*, *a_r_* the fraction of links that have one or both vertices inside the community *r*, and the sum extends to all communities *r* in a given network [30]. The modularity of a given partition is then the probability of having edges falling within groups in the network minus the expected probability in an equivalent (null case) network with the same number of nodes, and edges placed at random preserving the nodes' degree. There exist many other ways to introduce the concept of community that can be found in the literature of social sciences [14], we have chosen modularity for being a global observable proposed in the physics literature with a large success in the identification of known sub-structure in networks.

Among the wide variety of algorithms available to carry out this maximization process [31], we used a divisive algorithm proposed by one of the authors based on Extremal Optimization (EO) heuristics [32]. A detailed description of the method is beyond the scope of the paper, but full details can be found elsewhere [33]. Any other algorithm to optimize modularity can be used, provided the optimal values of modularity found are competitive.

Once we have determined the number and size of the network communities, we focus on the study of two structural mesoscopic characteristics: The connectivity between communities and their internal organization.

### Inter-community structure

**Figure 2:**
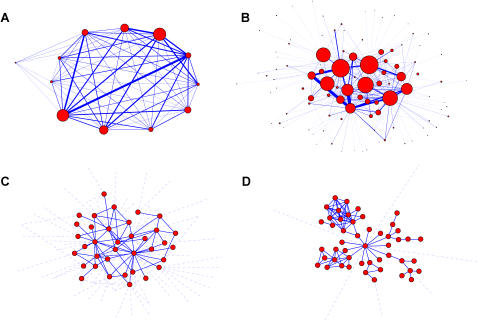
Community structures of the email and PGP networks. Top: Community structures of the email (A) and PGP (B) networks. Nodes correspond to communities (where size is proportional to their number of members) and links represent cross-connections (where width corresponds to the number of inter-connetions). Bottom: Typical examples of the communities detected in the email (C) and PGP (D) networks. Solid links join nodes of the community, dashed links join this community with others.

To summarize the results obtained from a community analysis of both social networks and to facilitate their comparison, the outcome of our analysis is jointly presented in Figure 2 (*A* and *B* for the email and PGP, respectively). Each node corresponds to a community, and a link between two nodes denotes cross-relations. In addition, the size of nodes and links gives information about community size and number of cross-links, respectively. It is evident from the plot that communities in the email network are densely interconnected, and sparsely interconnected in the PGP network. The calculation of the weighted degree distribution (the distribution of the sums of weights of links for each node) *P*(*ω*) confirms this evidence: the email community network has a 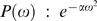 while the PGP community network presents a *P*(*ω*) : *e^−βω^*.

### Intra-community structure

The internal structure of communities in both networks also presents important differences. In Fig. 2*C* and Fig. 2*D* we plot the aspect of representative communities of the email and PGP networks, respectively. From the plot, the differences in the internal structure are clear: the email communities present a very homogeneous structure when compared with the heterogeneity of the PGP communities. For a more quantitative assesment of this difference, we have calculated the relative difference between the average and the maximum value of the internal degree in each community, Δ*H*. This measure allows to grasp the heterogeneity of the internal community structure. While Δ*H* : 5 in the email network, the values of Δ*H* in the PGP network range from 5 up to 35, confirming our observations. In the following, we will call *local hubs* the nodes in the PGP networks responsible for the very high Δ*H*≈30.

### Hypothesis

Previous works have stressed the role of hubs at a macroscopical level in PD dynamics on adaptive networks [3]–[37]. Although our networks are static, it is expected that the presence of these local hubs in PGP communities (as well as their absence in the email ones) influences strongly the evolution of the PD on these networks. To be specific, local hubs play a double stabilizing role: First, as most nodes in the community are directly linked to their local hub, the whole community tends to imitate the strategy of the hub; second, when a less connected member of the community changes her strategy following an external node, the influence of the local hub makes it harder for this strategy to spread to the whole community.

On the contrary, homogeneous internal degree distributions, as in the case of the email network, lead to a behavior that is not governed by hubs: All nodes are more or less equivalent, and indeed simulations show that their strategies evolve in a synchronized manner, at least to some degree. Therefore, the behavior of the email network will be more dependent on how the communities are connected among themselves. We thus are in a position to formulate our hypothesis: the behavior observed in a network with communities depends strongly on the intra-community heterogeneity (IH) and on the inter-community connectivity (IC). In this scenario, the robustness of cooperation observed in the PGP network is due to its low IC and high IH, whereas the fact that cooperation only arises for low *b* in the email network arises from its high IC and low IH.

## Discussion

### Test of our hypothesis in model networks

As we have seen, the analysis of the email and PGP networks raised two characteristic patterns of the mesoscale: (i) IH, or existence or not of local hubs in the network (Intra-Heterogeneity), and (ii) IC, the degree of connections between communities (Inter-Connectivity). To test this hypothesis, we propose to use synthetic networks as a benchmark in which to tune the above mechanisms as follows:

- First we divide a number of nodes *N*, into *m* communities of equivalent size.
- Second, we prescribe the IH. We have used as standard mechanisms for the construction of *ad hoc* homogeneous and heterogeneous communities the Erdos-Renyi model [38] and the heterogeneous (scale-free) random graph resulting from the Barabasi-Albert model [39], respectively. In the first case the probability of connection between two nodes is constant (p_intra_); in the second case, the network grows by adding nodes with *k_0_* links to an initial connected core, and the probability of connection of a node *i* to another existing node *j* is proportional to the current degree of node *j*.
- Third, we prescribe the IC. We construct a unique connected component by linking the communities previously generated. To interconnect the resulting communities we prescribe a new constant probability *p_inter_* to form links between two randomly selected nodes from the pool of communities, whenever these nodes below to different communities. The density of cross-connections is controlled by the probability *p_inter_*. Note that *p_inter_* must be sufficiently large to ensure the existence ofa unique connected component, but not so high as to mask the actual communities (i.e. an accurate detection algorithm should still separate the prescribed communities).
- Finally, we check by using a community detection algorithm (extremal optimization [33]) that the communities obtained at the best partition of modularity are the prescribed ones.

We have built up four statistically significant synthetic test networks with the same number of nodes (*N* = 10000) and the same number of communities (*m* = 75), corresponding to four extremal configurations corresponding to the combination of low and high values of the IH and IC. Our expectation is that the configuration corresponding to low IH and high IC will be representative of the class of mesoscopic traits observed in the email network; conversely, high IH and low IC should be representative of the class of mesoscopic traits observed in the PGP network. The other two cases, low IH and low IC, and high IH and high IC should constitute intermediate configurations between the former ones. The statistical properties of the so obtained networks are listed in Table 3.

**Table 3:** Statistical properties of synthetic networks with 10000 nodes and 75 communities.

Class	P(k)	〈C〉	r
A (Low IH – High IC)	∼exp (−0.0018*k^2^*)	0.031	0.013
B (Low IH – Low IC)	∼*k* ^∧^(−2.47) if *k*<30	0.040	−0.202
	∼*k* ^∧^(−0.047*k*) if *k*>30		
C (High IH – High IC)	∼exp (−0.003*k^2^*)	0.080	0.110
D (High IH – Low IC)	∼*k* ^∧^(−2.38) if *k*<30	0.090	−0.308
	∼*k* ^∧^(−0.027*k*) if *k*>30		

P(k) is the degree distribution (best fit to the data using least squares method), 〈C〉 is the clustering coefficient, and r stands for the assortativity coefficient [43]. See definition of these statistical descriptors in Table 2.

At this point, we want to emphasize that the statistical properties of the email and PGP networks (Table 2) and their synthetic counterparts (cases *A* and *D* in Table 3) present strong dissimilarities. First, we notice that the degree distributions of the synthetic networks are different from those observed in real networks. In addition, the clustering coefficient of the synthetic networks is almost an order of magnitude smaller than in the real networks. Finally, the assortativity coefficient of the synthetic class with low IC is negative, while the other two present positive values of assortativity. These different statistical properties of our synthetic and empirical networks are specially interesting for the validation process: Since the list of similarities between the two sets of networks has been reduced to the desired inter and intracommunity structural properties, any agreement we may find on the behavior of cooperation dynamics can be safely attributed to these mesoscopic features.

**Figure 3:**
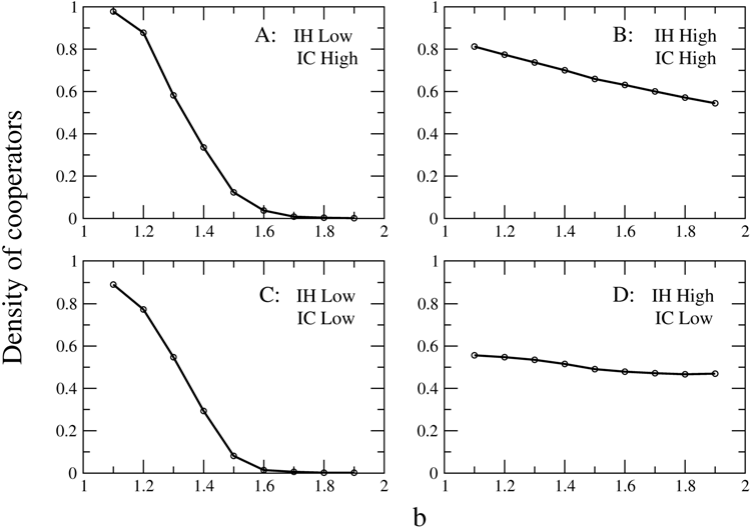
Evolution of cooperation in four synthetic networks. Cases A and D correspond, respectively, to the synthetic classes of networks akin to the email and PGP real networks. In case A communities have been built as Erdos-Renyi random graphs (p_intra_ = 1.5×10^−1^), and the probability of interconnection between communities (p_inter_) is 5×10^−2^. Communities in case D are constructed as independent scale-free networks (Barabasi-Albert with k_0_ = 3), and after they have been sparsely interconnected with (p_inter_ = 1.5×10^−5^). Case B has been obtained from D by increasing the probability p_inter_ to 3.5×10^−4^, and case C corresponds to A reducing this probability to 7.5×10^−4^. Simulations have been performed as indicated in Fig. 1.

Figure 3 shows the evolution of cooperation as a function of the temptation parameter *b* for our four synthetic networks. We discuss first the behavior of networks corresponding to A and D configurations (the *synthetic classes* of the empirical email and PGP networks, respectively). Although, in general, the values of final density of cooperators are smaller than those reached in the empirical cases (see Figure 1), synthetic networks reproduce the qualitative behaviors of the two real social networks in terms of sensitivity to changes of the temptation value *b*. Actually, the evolution of cooperation on the synthetic networks presents the observed tendencies even more emphasized than the empirical ones. On the one hand, all values of density of cooperators in case D (*PGP-class*) are close to the density established as initial fraction of cooperators (*ρ* = 0.5), revealing extraordinarily high levels of stability of the strategies played by agents. On the other hand, the decrease on the cooperation level shown in case A (*email-class*) is somewhat larger than that of the empirical email network for the same range of temptation values.

Additional plots in Fig. 3 help us to understand, separately, how each mesoscopic characteristic acts over cooperation. Comparing the behavior of configurations A and D with the other two classes, B and C, we observe that when both mesoscopic characteristics are high (configuration B), the system presents remarkable rates of cooperation. On the other hand, for low values of both mesoscopic characteristics (configuration C) the sensitivity to the temptation parameter is increased, the maximum level of cooperation is smaller, and the decay on cooperation is sharper. Consequently, we observe that IH seems to be more determinant than IC as a stabilizing factor against changes on the temptation to defect. We interpret this fact in agreement with macroscopic observations in scale-free networks where hubs play essentially this role [28]. In our mesoscopic description, a high IH always implies the existence of “local hubs”, that are hubs with respect to the rest of members of the community although they are not necessarily hubs at a global scale. These local hubs are the key nodes on which the robustness of the cooperative behavior relies and correspondingly IH is the magnitude informing of their existence. Conversely, IC is more relevant to achieve a larger level of cooperation than IH. Interestingly, if IC is set to zero, we end up with a set of disconnected networks where the level of cooperation will always be lower than if we connect the groups; this can be viewed as a positive reinforcement of cooperation between groups (communities). It is then clear that the behaviors observed on the email and PGP empirical networks (and on their synthetic counterparts) is the result of the interplay of both mesoscopic structural properties, since we cannot reach case D from case A by tuning only one of them.

For practical purposes, the computation of the value of IC and IH can be done *a posteriori* once the community structure of the network is obtained, the logical steps to their computation are then: i) Extract the community structure of the network using algorithms devised to this end (the more accurate its determination the more realible the values of IC and IH), ii) compute IC as the density of cross-connections between communities, and iii) compute IH as the average of the variance of the normalized degree whitin each community. A more detailed description of this procedure, including an example with a simple network (Figure S3), can be found in Text S1. We have tested this procedure on both our real networks and our synthetic models with quite satisfactory results. The values obtained using this procedure are collected in Table S1. Details on the procedure and an example of its application to a simple graph can be found in the Supplementary text. It is important to note that the two quantities we introduce can be computed for any graph and are therefore a systematic way to obtain quantitative information about its mesoscopic properties.

### Conclusions

In this work we have addressed the issue of the emergence of cooperation on true social networks in the framework of the evolutionary PD with imitation. Our results on two different networks show clearly that the specific details of the network considered are very relevant to determine the level of cooperation reached. Our analysis of the community structure of both networks lead us to the hypothesis that two mesoscopic structural properties (the connectivity between communities, IC, and their internal structure, IH) influence the evolution of cooperation in social networks by raising or lowering the level of cooperation and the stability of the behavior of the communities against changes on the temptation to defect. In order to verify this claim, we have designed synthetic model networks where these two features can be tuned as desired. Simulations on four such synthetic networks confirmed that, though their structural features have little in common with the empirical ones, except for the mesoscopic characteristics under study, the behavior of cooperation is very similar. Our models also show that both mesoscopic structural characteristics, IH and IC, influence the robustness of cooperation against changes on the temptation to defect, in excellent agreement with the observation made in the real social networks analyzed. Finally, we have introduced a procedure to obtain these two quantities quantitatively from any network.

We stress that, as stated in the introduction, our results combine two ingredients: locality (given by the network) and learning by imitation. In this paper we focus on the network structure and find uncontestable evidence of the relevance of IH and IC on the dynamics given by our update rule, unconditional imitation. This is enough to claim that network structure has to be taken into account in general, as aggregate characteristics may not give clues to understanding their behavior. However, we realize that the question then arises as to the influence of these network features on other dynamics. A thorough study of this issue is beyond the present work, because evolutionary game theory on graphs depends very strongly on the specific rule considered, and there are very many different choices [11]–[13]. In the case of the networks studied here, it is important to have in mind that unconditional imitation leads to *lower* levels of cooperation [13] than the stochastic rule used in [16] (proportional update). On the other hand, hubs have been shown recently to play a role similar to the one discussed here under such a proportional update dynamics [37]. To verify that our results are not an artifact of the imitation rule, we have repeated our simulations with proportional update. The results are qualitatively the same, the decrease in cooperation being more abrupt for the email network and somewhat steeper for the PGP network, but the simulations are much more demanding because it takes much longer to reach a steady state. Therefore, while a detailed comparison of these (and other) rules would require considerable computational effort, we can at least be sure that the general scenario we are describing will apply to proportional update. On the other hand, best-response-type rules lead, generally speaking, to the same outcome as well mixed populations [13], and it is clear that in that case the network structure might control the time to reach asymptotics, but not the final state itself. In any event, it is clear that this issue deserves further and thorough study.

Dwelling further on the evolutionary perspective, the work by Eguíluz et al. [3] indicates that if the network is allowed to co-evolve with the strategies, a network with hubs develops. Interestingly, in this network with hubs, the cooperation level shows similar dependence on the temptation parameter, much as we have found here for the PGP network. Along similar lines, recent work by Santos et al. [36], [40] suggests a connection between the emergence of cooperation and the evolutionary appearance of degree heterogeneity. In this context, our study, which we stress is carried out on static networks, suggests that the cooperation levels we observe in the PD may be related to the different origin of the two networks: While the PGP network is spontaneously formed and with a clearly cooperative goal in mind (namely, finding help to ensure communication privacy), the email network arises from an underlying external structure, whose main purpose is not so clearly cooperative as it involves many other aspects and tasks. Our results would then support the existence of community structures organized around hubs with resilient cooperative behavior.

The above comment suggests, in addition, that our results may be of interest for the design of hierarchies and organizations with tailored cooperation behavior. We have seen that the email network reaches, for moderate values of the temptation parameter, cooperation levels very close to the optimum. Therefore, networks with this structure should be used in order to achieve very high performance levels in terms of cooperation. On the other hand, while the email network is quite susceptible to an increase of the temptation parameter, and hence exhibits a degrading of the cooperation for large temptations, the PGP network, with its weakly connected communities with hubs, is much more robust in this respect, and ensures cooperation for almost any temptation. Organizations with a PGP-like structure would exhibit a very robust cooperation, although there would always be defectors. In connection with this, it is important to note that our social networks are obtained by looking at bidirectional links, which may be related to an a priori willingness to cooperate among the linked individuals. This may be an important ingredient for the design of cooperative networks and a hint towards the understanding of cooperation. Further research at the mesoscopic scale, looking at different combinations of IH and IC structures, could lead to designs that would be both optimal and robust (such as, e.g., the structure corresponding to Fig. 3B). Interestingly, this conclusion may carry over to different dynamical contexts (other than evolutionary game theory): For instance, recent results on synchronization dynamics in a system of coupled oscillators show a strong influence of the community structure as well [41], and hence communities have to be taken into account much in the same way we are describing here. On the other hand, an intriguing issue is the connection of our results to the problem of the evolutionary origin of cooperation. One of the explanations suggested in this framework is the relevant role of group selection (see, e.g., [42] and references therein). In this context, the possibility of making a connection between communities and group-like entities seems very appealing, and is certainly a topic worth pursuing.

Finally, we want to emphasize our main conclusion, namely that cooperation in real social networks is a complex issue depending on the combination of the effects of several structural features. This result has far-reaching implications: Thus, several previous researches have considered how cooperation emerges in the PD on different model networks, including gaussian, scale free and small world ones as paradigms of social networks. There are two main differences between our work and those previous ones: first, the cooperation level is in general higher that in the model networks, and second, results are very different for similar global parameters of the network due to the influence of the community structure, often undetected by global measurements. It is then clear that any approximation to the evolution of cooperation in social networks based on the generalization of only one of these structural features is far too simplistic and may be misleading. Although, as stated in the introduction, we are studying here the hardest social dilemma, we envisage that similar conclusions may apply to the other dilemmas represented by coordination or anti-coordination games, as arguments based on the inter- and intra-structure of the communities may well carry over to them. In any event, we believe that subsequent studies on these issues should then be carried out on a case by case basis, and should involve a careful analysis at a mesoscopic (community) level, trying to find out whether behaviors can be predicted or classified in classes attending to this structure.

## Supporting Information

Figure S1 Sensitivity to different initial conditions. For both the two empirical networks under study (email and PGP), the plots show the final density of cooperators as ain function of b for different initial proportions of cooperators (Co). Plots in black (Co = 0.5) correspond to the results shown in Fig. 1. Significantly, the PGP network presents a more stablegeneric behavior for a wide range of initial scenarios ranging from Co = 0.3 to Co = 0.7, supporting the robustness of the results reported in the paper. The equilibrium densities of cooperators have been obtained by averaging 500 generations, after a transient time of 750 generation steps. Each point has been averaged over 1000 independent simulations.
(1.72 MB TIF)

Figure S2 Sensitivity to positive values of the Punishment (P) payoff. For two networks corresponding to configurations A and D (the synthetic classes of the empirical substrates), the plots show the density of cooperators in as a function of b obtained using two alternative definitions ofdifferent sets of payoffs for the Prisoner's Dilemma game. Black lines correspond to simulations preserving with the game definitionsame payoffs as in the paper (T = b, R = 1, P = S = 0), while red lines stand for a definition with a positive P value (T = b, R = 1, P = 0.01, S = 0). We observe that the results presented in the paper do not change significantly when P>0, i.e., when we are in the pure Prisoner's Dilemma and away from its boundary with the Snowdrift game (corresponding to P<0). The equilibrium densities of cooperators have been obtained by averaging 500 generations, after a transient time of 750 generation steps. Each point corresponds to an average over 1000 independent simulations with 50% cooperators and defectors as the initial condition.
(8.96 MB TIF)

Figure S3 Toy model network to illustrate the computation of IH and IC. In Text S1, we use this network to provide a simple example of quantification of IH and IC. Dashed lines correspond to cross-links between the two different communities.
(0.73 MB TIF)

Table S1 Values of IH (Intra-community Heterogeneity) and IC (Inter-community Connectivity) for all the networks used in the manuscript (both empirical and synthetic). The procedure followed to obtain these quantities is explained in Text S1. These values confirm quantitatively what was already expressed qualitatively along the text: On one side, email communities are less heterogeneous and more densely interconnected than PGP ones. On the other side, the synthetic networks (and, particularly, configurations A and D), represent extreme cases.
(0.05 MB DOC)

Text S1 Procedures to compute Intra-community Heterogeneity (IH) and Inter-community Connectivity (IC).
(0.16 MB PDF)
