# Supplementary figures and images for: Mesoscopic Structure Conditions the Emergence of Cooperation on Social Networks
Source: PLoS One. 2008 Apr 2;3(4):e1892. doi: 10.1371/journal.pone.0001892 (PMC2274863; doi:10.1371/journal.pone.0001892)

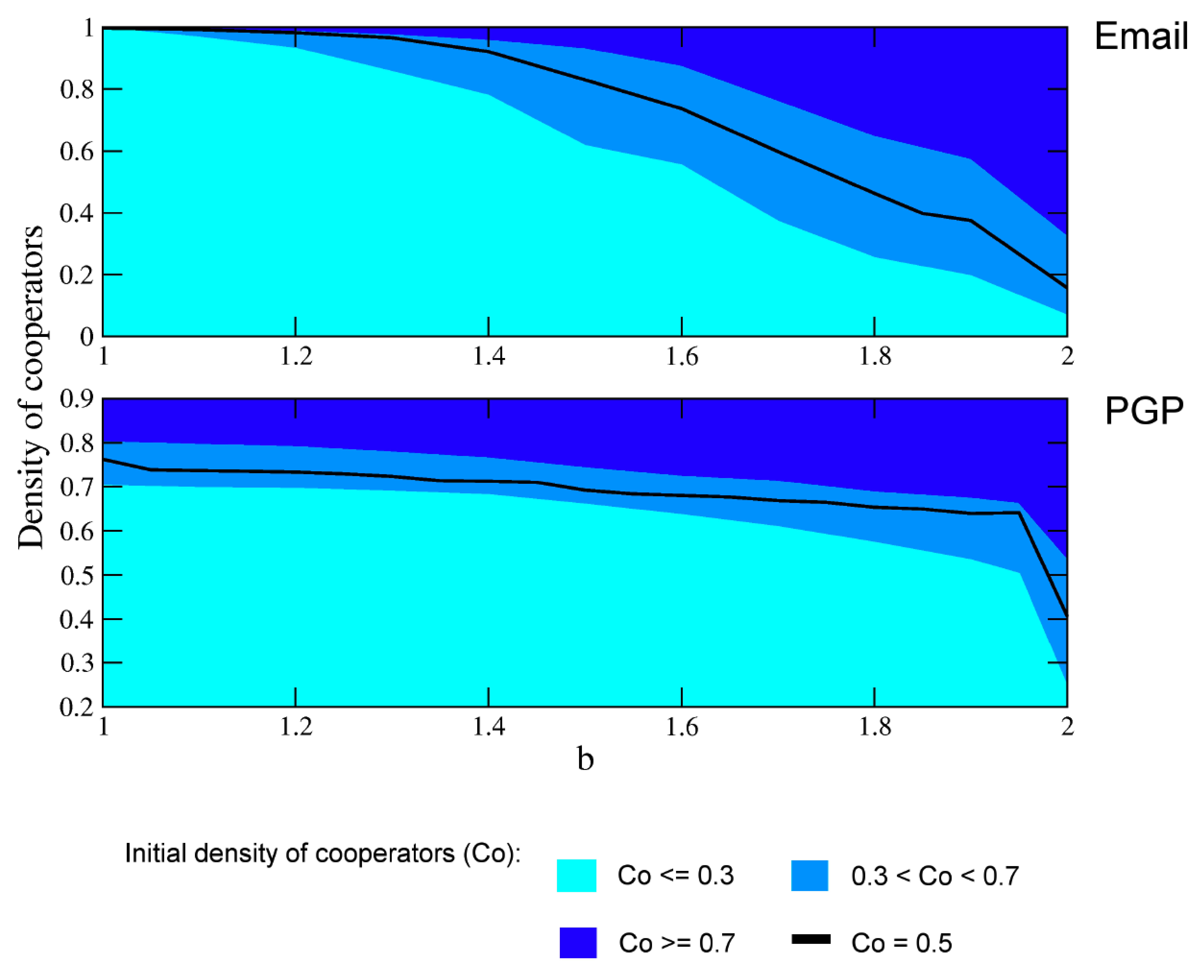

Supplement: Figure S1 — Sensitivity to different initial conditions. For both the two empirical networks under study (email and PGP), the plots show the final density of cooperators as ain function of b for different initial proportions of cooperators (Co). Plots in black (Co = 0.5) correspond to the results shown in Fig. 1. Significantly, the PGP network presents a more stablegeneric behavior for a wide range of initial scenarios ranging from Co = 0.3 to Co = 0.7, supporting the robustness of the results reported in the paper. The equilibrium densities of cooperators have been obtained by averaging 500 generations, after a transient time of 750 generation steps. Each point has been averaged over 1000 independent simulations. (1.72 MB TIF) [file pone.0001892.s001.tif]

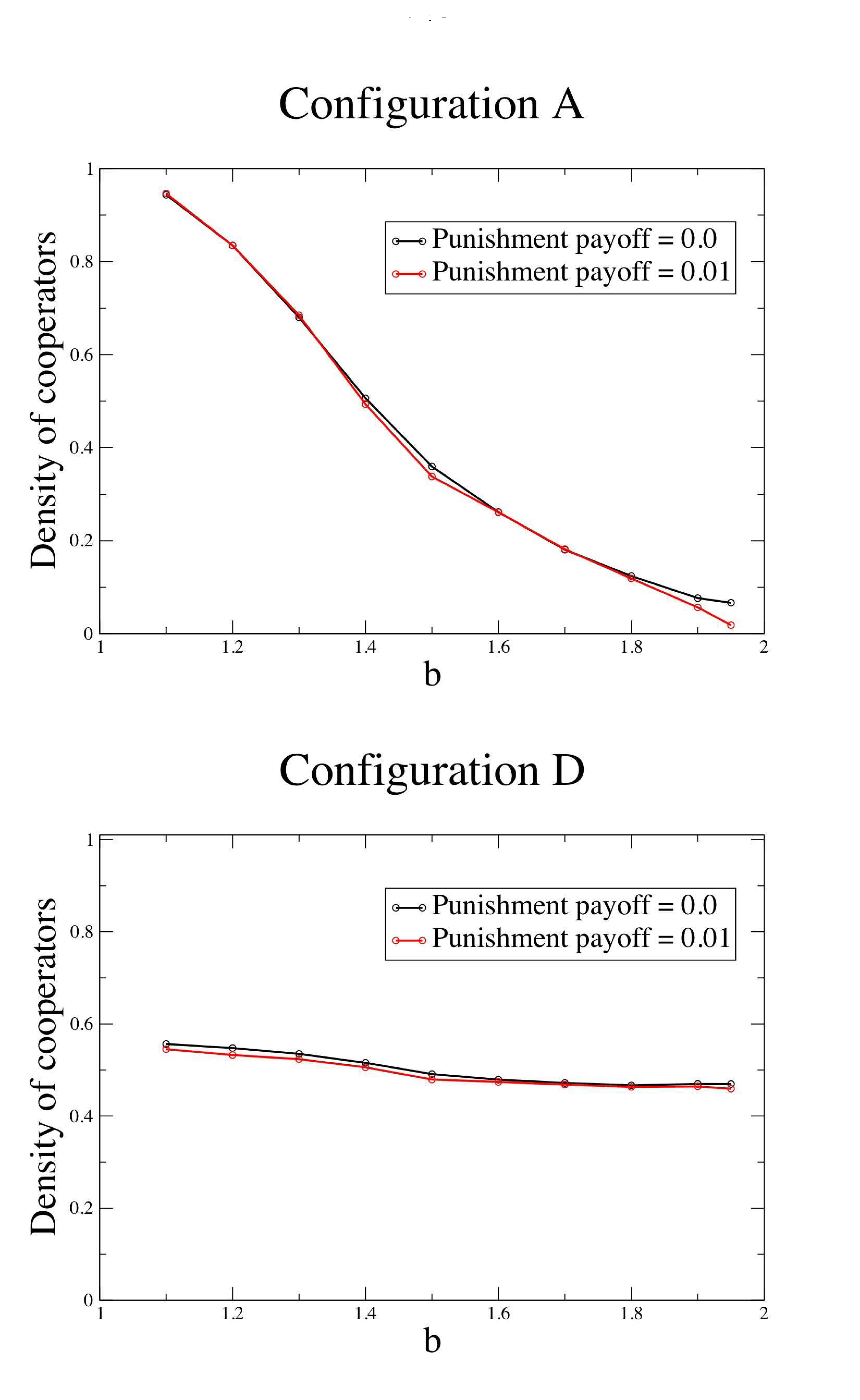

Supplement: Figure S2 — Sensitivity to positive values of the Punishment (P) payoff. For two networks corresponding to configurations A and D (the synthetic classes of the empirical substrates), the plots show the density of cooperators in as a function of b obtained using two alternative definitions ofdifferent sets of payoffs for the Prisoner's Dilemma game. Black lines correspond to simulations preserving with the game definitionsame payoffs as in the paper (T = b, R = 1, P = S = 0), while red lines stand for a definition with a positive P value (T = b, R = 1, P = 0.01, S = 0). We observe that the results presented in the paper do not change significantly when P>0, i.e., when we are in the pure Prisoner's Dilemma and away from its boundary with the Snowdrift game (corresponding to P<0). The equilibrium densities of cooperators have been obtained by averaging 500 generations, after a transient time of 750 generation steps. Each point corresponds to an average over 1000 independent simulations with 50% cooperators and defectors as the initial condition. (8.96 MB TIF) [file pone.0001892.s002.tif]

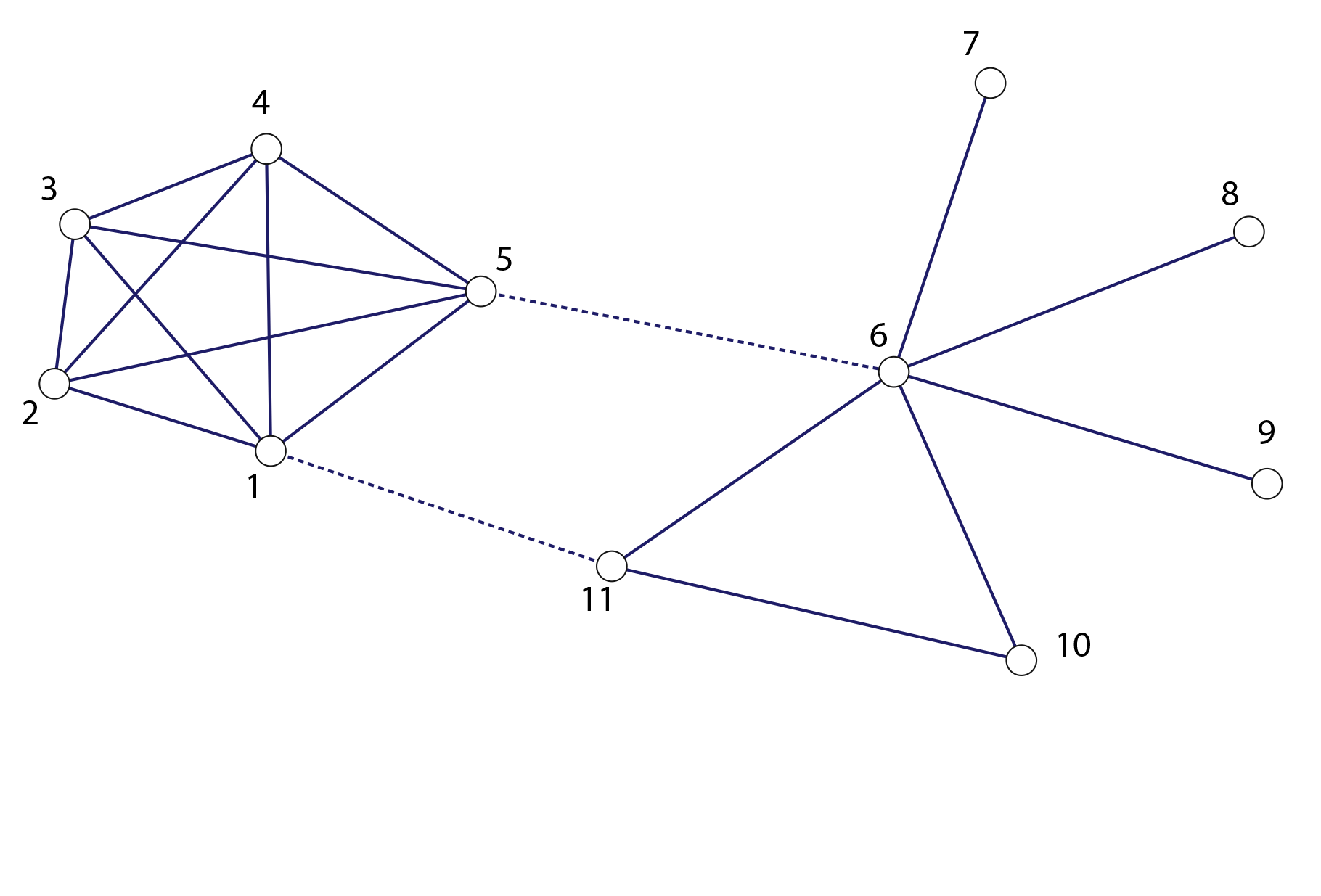

Supplement: Figure S3 — Toy model network to illustrate the computation of IH and IC. In Text S1, we use this network to provide a simple example of quantification of IH and IC. Dashed lines correspond to cross-links between the two different communities. (0.73 MB TIF) [file pone.0001892.s003.tif]
