# Supplementary material for: Mesoscopic Structure Conditions the Emergence of Cooperation on Social Networks
Source: PLoS One. 2008 Apr 2;3(4):e1892. doi: 10.1371/journal.pone.0001892 (PMC2274863; doi:10.1371/journal.pone.0001892)
Supplement: Table S1 — Values of IH (Intra-community Heterogeneity) and IC (Inter-community Connectivity) for all the networks used in the manuscript (both empirical and synthetic). The procedure followed to obtain these quantities is explained in Text S1. These values confirm quantitatively what was already expressed qualitatively along the text: On one side, email communities are less heterogeneous and more densely interconnected than PGP ones. On the other side, the synthetic networks (and, particularly, configurations A and D), represent extreme cases. (0.05 MB DOC) [file pone.0001892.s004.doc]

**Table S1: Values of IH (Intra-community Heterogeneity) and IC (Inter-community Connectivity) for all the networks used in the manuscript (both empirical and synthetic).**

| **Network** | **IH value** | **IC value** |
| --- | --- | --- |
| email | 0.75 |  |
| PGP | 1.36 |  |
| Configuration A | 0.38 |  |
| Configuration B | 0.52 |  |
| Configuration C | 1.81 |  |
| Configuration D | 2.10 |  |

The procedure followed to obtain these quantities is explained in Text S1. These values confirm quantitatively what was already expressed qualitatively along the text: On one side, email communities are less heterogeneous and more densely interconnected than PGP ones. On the other side, the synthetic networks (and, particularly, configurations A and D) represent extreme cases.
