# Supplementary material for: Mesoscopic Structure Conditions the Emergence of Cooperation on Social Networks
Source: PLoS One. 2008 Apr 2;3(4):e1892. doi: 10.1371/journal.pone.0001892 (PMC2274863; doi:10.1371/journal.pone.0001892)
Supplement: Text S1 — Procedures to compute Intra-community Heterogeneity (IH) and Inter-community Connectivity (IC). (0.16 MB PDF) [file pone.0001892.s005.pdf]

Procedures to compute Intra-community Heterogeneity (IH) and Inter-community Connectivity (IC).

For the calculation of IH and IC values presented in Table S1, we have used the following procedures:

<u>Calculation of IH</u>

1. Extract the community structure of the network using algorithms devised to this end.

2. For each community:

    a. Calculate the internal degree of each node (that is, the number of links to other nodes belonging to the same community).
    b. Obtain the average internal degree of the community $\langle k_{\mathrm{int}ra} \rangle$.
    c. Calculate the standard deviation of the internal degree normalizing by the average value:

$$\sigma_{\mathrm{int}ra} = \sqrt{\frac{\sum_{i \in community} \left(\frac{(k_{\mathrm{int}ra})_i - \langle k_{\mathrm{int}ra} \rangle}{\langle k_{\mathrm{int}ra} \rangle} \right)^2}{N_{community}}}$$

    This normalization facilitates the comparison between the standard deviation of communities with different sizes and average internal degrees.

3. Compute IH as the average over communities of the standard deviation.

<u>Calculation of IC</u>

1. Extract the community structure of the network using algorithms devised to this end.

2. For each node:

    a. Count the number of cross-connections (links connecting to nodes belonging to other communities).
    b. Compute the fraction of cross-connections as the ratio of the quantity obtained in the previous step to the maximum possible number of cross-connections (that is, the community population times the rest of the population in the network):

3. Compute IC as the average over all nodes of the fraction of cross-connections.

Example: a toy model

In order to make these procedures clear, in the following we present the calculation of IH and IC values for a very simple network (see Fig. S3). The sample network is composed by two communities completely different in terms of internal structure. One of the communities is a

completely homogeneous clique (community 1), while the other one presents a star-like topology (community 2).

<u>Calculation of IH</u>

Community 1:

$$\langle k_{\text{int}\,ra} \rangle_1 = \frac{5 \cdot 4}{5} = 4$$

$$(\sigma_{\text{int}\,ra})_1 = \sqrt{\frac{5 \cdot \left(\frac{4-4}{4} \right)^2}{5}} = 0$$

Community 2:

$$\langle k_{\text{int}\,ra} \rangle_2 = \frac{1 \cdot 5 + 2 \cdot 2 + 3 \cdot 1}{6} = \frac{12}{6} = 2$$

$$(\sigma_{\text{int}\,ra})_2 = \sqrt{\frac{1 \cdot \left(\frac{5-2}{2} \right)^2 + 2 \cdot \left(\frac{2-2}{2} \right)^2 + 3 \cdot \left(\frac{1-2}{2} \right)^2}{6}} = \sqrt{\frac{\frac{9}{4} + \frac{1}{4}}{6}} = \sqrt{\frac{5}{12}} = 0.645$$

Finally, IH can then be obtained as the average over the communities:

$$\boxed{IH = \frac{0 + 0.645}{2} = 0.3225}$$

Calculation of IC

Only 2 nodes in each community have cross-interactions: 5 and 1 (community 1) and 6 and 11 (community 2). Consequently, the calculation of the fraction of cross-connections is the following:

Nodes 1 and 5: $\frac{1}{N_{nodes\_community2}} = \frac{1}{6}$

Nodes 6 and 11: $\frac{1}{N_{nodes\_community1}} = \frac{1}{5}$

Rest of nodes: 0

IC is now the average over all nodes in the network:

$$IC = \frac{0 + 2 \cdot \frac{1}{6} + 2 \cdot \frac{1}{5}}{11} = 0.066$$
